# Supplementary material for: Causal Relationship between Aging and Anorexia Nervosa: A White-Matter-Microstructure-Mediated Mendelian Randomization Analysis
Source: Biomedicines. 2024 Aug 16;12(8):1874. doi: 10.3390/biomedicines12081874 (PMC11351342; doi:10.3390/biomedicines12081874)
Supplement: Supplementary file 1 [file biomedicines-12-01874-s001.zip › Supplementary Figure/Figure S4/Singe SNP plot of MR analysis for TL to ubm-b-2034.pdf]

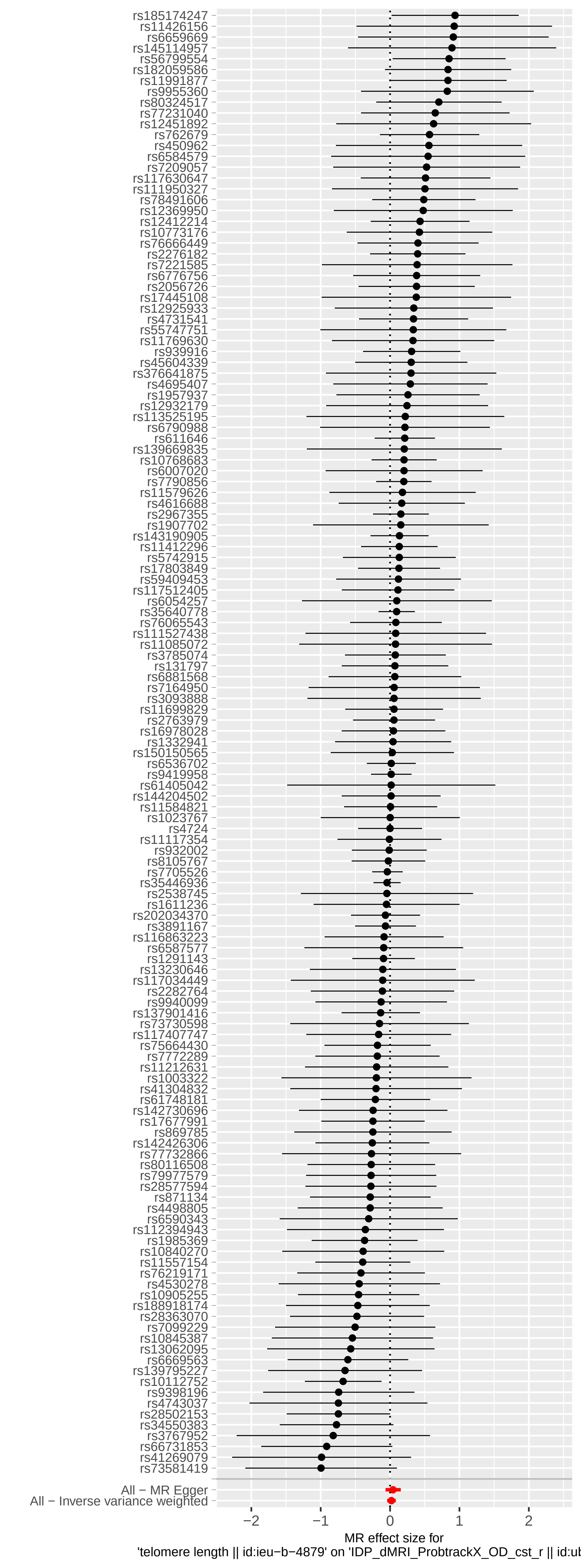
rs185174247
rs11426156
rs6659669
rs145114957
rs56799554
rs182059586
rs11991877
rs9955360
rs80324517
rs77231040
rs12451892
rs762679
rs450962
rs6584579
rs7209057
rs117630647
rs111950327
rs78491606
rs12369950
rs12412214
rs10773176
rs76666449
rs2276182
rs7221585
rs6776756
rs2056726
rs17445108
rs12925933
rs4731541
rs55747751
rs11769630
rs939916
rs45604339
rs376641875
rs4695407
rs1957937
rs12932179
rs113525195
rs6790988
rs611646
rs139669835
rs10768683
rs6007020
rs7790856
rs11579626
rs4616688
rs2967355
rs1907702
rs143190905
rs11412296
rs5742915
rs17803849
rs59409453
rs117512405
rs6054257
rs35640778
rs76065543
rs111527438
rs11085072
rs3785074
rs131797
rs6881568
rs7164950
rs3093888
rs11699829
rs2763979
rs16978028
rs1332941
rs150150565
rs6536702
rs9419958
rs61405042
rs144204502
rs11584821
rs1023767
rs4724
rs11117354
rs932002
rs8105767
rs7705526
rs35446936
rs2538745
rs1611236
rs202034370
rs3891167
rs116863223
rs6587577
rs1291143
rs13230646
rs117034449
rs2282764
rs9940099
rs137901416
rs73730598
rs117407747
rs75664430
rs7772289
rs11212631
rs1003322
rs41304832
rs61748181
rs142730696
rs17677991
rs869785
rs142426306
rs77732866
rs80116508
rs79977579
rs28577594
rs871134
rs4498805
rs6590343
rs112394943
rs1985369
rs10840270
rs11557154
rs76219171
rs4530278
rs10905255
rs188918174
rs28363070
rs7099229
rs10845387
rs13062095
rs6669563
rs139795227
rs10112752
rs9398196
rs4743037
rs28502153
rs34550383
rs3767952
rs66731853
rs41269079
rs73581419
All − MR Egger
All − Inverse variance weighted
−2
−1
0
1
2
MR effect size for
'telomere length || id:ieu−b−4879' on 'IDP_dMRI_ProbtrackX_OD_cst_r || id:ul
